# Selecting for CRISPR-Edited Knock-In Cells

**DOI:** 10.3390/ijms231911919

**Published:** 2022-10-07

**Authors:** Nina Reuven, Yosef Shaul

**Affiliations:** Department of Molecular Genetics, Weizmann Institute of Science, Rehovot 76100, Israel

**Keywords:** CRISPR, genome editing, HDR-dependent editing, scarless selection, co-editing

## Abstract

CRISPR technology affords a simple and robust way to edit the genomes of cells, providing powerful tools for basic research and medicine. While using Cas9 to target a genomic site is very efficient, making a specific mutation at that site is much less so, as it depends on the endogenous DNA repair machinery. Various strategies have been developed to increase the efficiency of knock-in mutagenesis, but often the desired cells remain a small percentage of the total population. To improve efficiency, strategies to select edited cells have been developed. In some applications, a selectable foreign gene is linked directly to the gene of interest (GOI). Alternatively, co-editing, where the GOI is edited along with a selectable gene, enriches the desired cells since the cells that successfully edited the selectable gene are likely to have also edited the GOI. To minimize perturbations of the host genome, “scarless” selection strategies have been developed, where the modified cells are mutated solely in the GOI. In this review, we will discuss strategies employed to improve specific genome editing in mammalian cells, focusing on ways to select successfully edited cells.

## 1. Introduction

CRISPR (Clustered regularly interspaced short palindromic repeats) is an elegant system for making DNA double-strand breaks (DSB) in a highly efficient and specific manner. Originating in the bacterial world, where it functions to protect bacteria from invading phages, the system has been leveraged to provide the first step in the genome engineering of organisms from microbes to humans [1,2,3,4]. The most commonly used components of the system are Cas9, having endonuclease functions, and the associated guide RNA (gRNA), which dictates where the Cas9 will bind and cleave the DNA. To simplify the system, the original two-part Cas9-associated RNAs, the CRISPR RNA (crRNA) and trans-activating CRISPR RNA (tracrRNA), are expressed as a fused single guide RNA (sgRNA), having both the programmable part for identifying the targeted locus and the tracrRNA portion for association with the Cas9 [2].

CRISPR technology provides a very efficient and easy-to-use method for targeting a specific locus in the genome. However, since editing of that locus depends on the endogenous DNA repair pathways, making a specific edit (a “knock-in” mutation) is much less efficient than using CRISPR to make a functional knock-out. Repair of a DSB by nonhomologous end joining (NHEJ) often results in small insertions or deletions (indels) at the site of the break (reviewed in [5]). This type of repair can lead to a frameshift, resulting in a knock-out mutation. In contrast, making a specific, template-directed mutation at a DSB requires homology directed repair (HDR), which is active during the S-G2 phases of the cell cycle [6,7,8]. Since NHEJ predominates over HDR in most cells [5], knock-in editing is not very efficient, and, therefore, different strategies have been developed to improve HDR-mediated gene editing (reviewed in [9]). 

## 2. Strategies to Increase HDR-Dependent CRISPR-Cas9 Mediated Genome Editing

### 2.1. Inhibiting NHEJ/Promoting HDR Globally to Increase CRISPR Knock-In Editing

**Figure 1:**
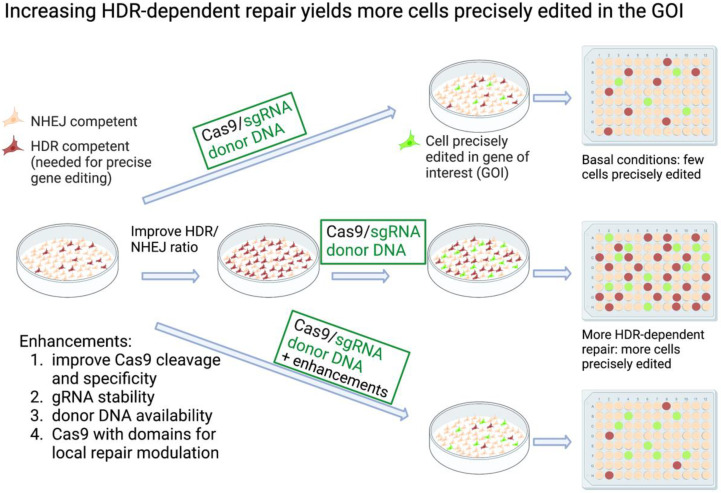
Increasing HDR-dependent repair improves yield of CRISPR knock-in cells. Knock-in HDR-dependent mutagenesis at a gene of interest (GOI). At the top, the basal conditions are shown, where few of the cells are HDR-competent. A fraction of these can be edited at the GOI following transfection with Cas9/gRNA and donor DNA. Isolating the desired knock-in edited cells from the total population can be challenging. In the center, cells are treated either genetically or pharmacologically to alter the HDR/NHEJ ratio. As a result, a higher yield of knock-in edited cells is achieved. The bottom row illustrates some methods used to improve HDR-dependent CRISPR editing. The increases in precisely edited cells depend on the method, the target cells and GOI. Created with BioRender.com.

Several of the approaches to increasing HDR-dependent gene editing involve shifting the balance between HDR and NHEJ (reviewed in [10,11,12]). Inhibiting NHEJ or promoting HDR, either genetically or pharmacologically, leads to increases in knock-in efficiency [13,14,15,16,17,18] (Figure 1, middle panel). Likewise, restricting Cas9 activity or expression to the S-G2 phases of the cell cycle, when HDR is active, also improves the knock-in yield (a comprehensive review of this topic can be found in [19]). Some of these methods include synchronizing the cells by treating them with drugs such as nocodazole (inhibitor of microtubule polymerization) [20] or the cyclin-dependent kinase inhibitor indirubin [21]. Another efficient method to restrict Cas9 activity to the S-G2 phases of the cell cycle is by fusing Cas9 to a fragment of a cell cycle-regulated protein, such as geminin [22]. Geminin is targeted for proteasomal degradation by the cell cycle-regulated APC-Cdh1, resulting in low levels in the G1 phase of the cell cycle and high levels during S/G2/M. 

### 2.2. Improving Cas9 Cleavage and Specificity

The Cas9 endonuclease most commonly used in genome engineering is derived from *Streptococcus pyogenes* (SpCas9). While it is efficient and easy to use, Cas9 from other sources or Cas9 which has been improved through rational design or directed evolution can offer advantages (reviewed in [12] (Figure 1, lower panel). For example, newer high-fidelity versions of Cas9 [23,24,25,26] reduce the possibility of off-target cutting. SpCas9 generates a blunt-ended DSB [2], which, as mentioned above, is predominantly processed by NHEJ. To produce a staggered cut that lends itself to precise editing via NHEJ [27], alternate programmable nucleases such as Cpf1 can be utilized [28]. Another way to improve specificity and produce a staggered cut is to use a dimeric RNA-guided FokI nuclease (RFN) [29] or fCas9 [30] comprised of a pair of catalytically dead Cas9, dCas9, fused to FokI. By using pairs of gRNAs to target the desired locus, the two FokI domains cleave the DNA according to the programmed spacing, producing a staggered cut. Because two gRNAs are required for targeting, specificity is increased. 

### 2.3. Modifications of the Guide RNA and Donor DNA to Improve Editing Efficiency

Another way to improve editing efficiency is to use modified gRNA (for a comprehensive review of this topic, see [31]) (Figure 1, lower panel). Chemically modified gRNA can serve to stabilize the gRNA by inhibiting its degradation and can improve the association of the tracrRNA with the crRNA [32,33]. Modification of the gRNA can also be used to improve HDR by bringing the donor DNA in close proximity to the cut site. In a method conceptually similar to the pegRNA described below, Lee et al. designed a unique RNA–DNA hybrid, where the RNA comprises the sgRNA and the DNA serves as donor [34]. The use of this construct increases HDR editing three-fold. In this work, they also demonstrated that HDR is improved two-fold by using a fluorescently labeled donor DNA and enriching the cells that took up the donor DNA by FACS sorting. The Marson lab has developed improved methods for the efficient editing of primary human T cells using non-viral genome targeting, finding ssDNA templates preferable to linear dsDNA, due to reduced toxicity and the chance of random integration [35,36]. A hybrid ssDNA template with dsDNA ends used for recruiting Cas9 further increased the yield of correctly modified cells, with knock-in efficiencies of up to 62%.

### 2.4. Promoting HDR Specifically at the Break Site

Recruiting HDR improving factors to the break site is another way to increase the yield of knock-in mutations (Figure 1, lower panel). HDR effectors such as CtIP, Rad52, or Mre11 fused to Cas9 promote HDR two-fold [37]. Another strategy implemented by Tran et al. is to use CtIP fused to the MS2 phage coat protein. This enables recruitment of the CtIP-MS2 to the cut site via MS2 binding sites on an extension of the gRNA. Since Cas9 is a rather large protein, approximately 160 kDa, fusing large domains to it can limit expression options, such as vectors with a limited payload. Using smaller functional domains, or recruiting strategies such as the MS2 loops, can avoid these issues. Fusion of the CtIP N-terminal 296 aa fragment to Cas9, for recruitment of HDR effectors, improves HDR-dependent editing by two-fold or more [38]. Two-fold improvement in HDR editing was also achieved with Cas9 fused to a 126 aa recruiting domain for the MRN complex (Mre11/Rad50/Nbs1) that is responsible for DNA resection needed for HDR. In this instance, the domain is derived from the HSV-1-encoded protein UL12 [39]. Alternatively, the fusion of Cas9 to a 413 aa dominant-negative 53BP1 fragment, DN1S, [40] serves to inhibit NHEJ locally at the targeted break site, which avoids potential random mutations caused by global inhibition of NHEJ. Several of these strategies were compared for their ability to edit the *HBB* (hemoglobin subunit beta) locus in human hematopoietic stem and progenitor cells (HSPCs) [41], confirming that the use of these modified Cas9 constructs can improve HDR-dependent editing in this system by two-fold. The *HBB* gene is mutated in sickle cell anemia and beta-thalassemia; thus, finding potent methods to effectively repair the mutation in patient-derived stem cells would have clear clinical benefits. Although doubling the yield of correctly edited cells may suffice for certain targets and cell lines, for most systems, more work needs to be conducted to optimize the overall yield of the correctly edited cells, especially regarding clinical applications.

### 2.5. Editing without a DSB—Circumventing the HDR Requirement

The repair of a DSB often generates undesired complications, as discussed above, so alternative strategies have been developed where the DSB is avoided altogether. These methods allow certain knock-in mutations to be made without the need for HDR. Base editing is a method using a catalytically inactive Cas9 fused to a base editor to accomplish specific base substitutions at the targeted site [42]. This method is efficient, with 15–75% editing success, but is limited to point mutations. Prime editing (PE) enables the insertion of longer fragments through the use of a catalytically impaired (nickase) Cas9 fused to a reverse transcriptase [43]. The donor template sequence is fused to the end of the gRNA (prime editing guide RNA, pegRNA), and the reverse transcriptase creates the donor DNA locally at the targeted site. As with base editing, this strategy avoids many of the pitfalls associated with the repair of DSBs and has an efficiency comparable to the HDR-dependent editing [43]. However, this method too is restricted to shorter insertions that can be encoded by the pegRNA. To improve upon this method, enabling all the components to be delivered by a single vector, Wolff et al. developed piggyPrime, a transfected single vector system based on piggyBac DNA transposition for genomic integration of all prime editing components [44]. In this system, the editing components (Cas9, etc.) remain integrated in the genome; thus, this system is not scarless. Eggenschwiler et al. presented a similar system, but with the ability to excise the prime editing components following the editing step using the piggyBac system for scarless editing [45]. 

## 3. Selecting Edited Cells

### 3.1. Identifying and Selecting for CRISPR-Edited Cells

Given that the CRISPR-edited knock-in cells are often a small percentage of the population, how can we identify, enrich, and isolate this population? In cases where the mutation in the GOI itself provides a selectable marker that can be used to identify and select the edited cells, the task is fairly simple. However, for mutated cells that are not phenotypically different from the unedited cells, this presents a challenge. Isolating and characterizing properly edited cells can be time-consuming and labor-intensive, as illustrated in Figure 1, where many individual colonies must be screened in order to identify the precisely edited cells. To improve upon this procedure, Miyaoka et al. developed a method using droplet digital PCR to identify properly edited cells harboring a point mutation within a population [46]. Using a strategy similar to sib-selection in yeast [47], a population of cells is subdivided, and the sub-population of cells harboring the proper mutation is further subdivided and analyzed until the desired cells are cloned. This method enables the isolation of specifically modified cells without antibiotic selection. The shortcomings of this approach are that several rounds of dilution and replating may be required before the desired clone is isolated, and it depends on developing a PCR assay that can reliably distinguish between the wild-type and mutant cells. 

Since identifying a rare subset of cells can be laborious, it is best if the starting population is enriched for the mutagenized cells. A basic approach to this problem is to enrich cells that express the Cas9 needed for editing. Using Cas9 tagged or connected via a ribosome-skip 2A peptide to a fluorescent protein or antibiotic resistance gene allows the selection of cells expressing Cas9 by FACS (fluorescence-activated cell sorting) or by antibiotic resistance [48,49]. For difficult-to-transfect cells, this enrichment is significant. However, as most of the repair of the Cas9-induced DSBs in this population is likely to be by NHEJ, the desired knock-in mutation cells requiring HDR will be rare. The challenge is to find a method that would specifically select cells that have undergone the desired edit in the gene of interest (GOI). The selection schemes can be divided into two main categories: (1) direct incorporation of a selectable marker into the edited GOI or (2) co-editing of a selectable marker together with the GOI. The second option is viable because of the possibility of CRISPR multiplexing [4], where several genes are edited simultaneously within a single cell. The selection strategies we describe below are those that specifically identify cells that have undergone knock-in editing. 

### 3.2. Selection Marker Expressed with Gene of Interest 

One use of CRISPR technology is to tag endogenous proteins with fluorescent or other tags to facilitate monitoring of the abundance, dynamics, and localization of endogenous proteins. Stewart-Ornstein and Lahav developed a gene-tagging toolkit (eFlut), providing template donor DNA plasmids for adding tags to endogenous genes, and incorporating additional antibiotic-resistance genes as selectable markers, separated by “self-cleaving” ribosome skip 2A peptides [50]. Properly edited cells can be easily selected by using the antibiotic. Alternatively, when using a simple construct only for fluorescent tagging, FACS-sorting can be employed to isolate the edited cells. The advantage to this method is that it is simple, employing short homology arms and a PCR-generated donor, and that the selection can quickly retrieve properly edited cells, even if they are a low percentage of the total cell population. It is possible to work directly with the pool of edited cells or to isolate single cell clones to more precisely characterize the genotype (for example, to verify if the editing was biallelic and, if not, determine what happened to the other allele). The shortcomings of this approach are: (1) it is limited to tagging at the N- or C-termini of the protein of interest; (2) the promoter of the GOI must be constitutively and strongly expressed to provide for the expression of the selectable marker gene; (3) the insertion of the selectable gene alters the length and composition of the mRNA, which could affect expression, stability, export, etc.

**Figure 2:**
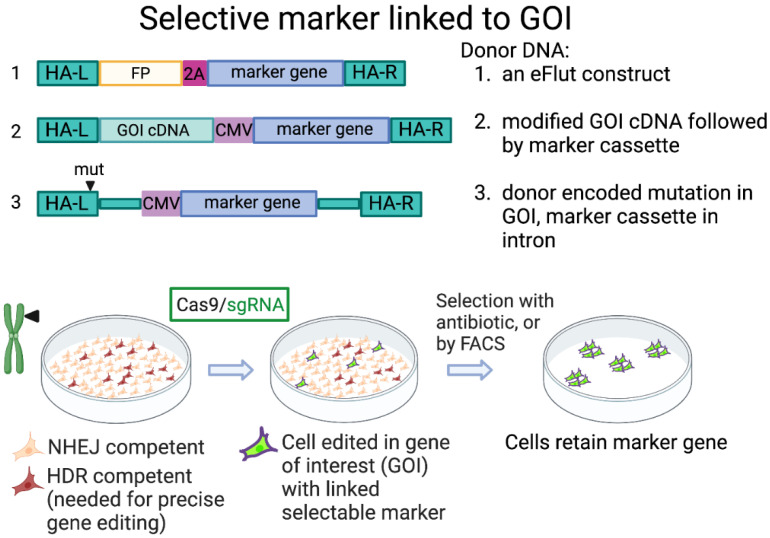
Selection marker linked to gene of interest. The selectable gene is linked directly, or through a 2A peptide, to the gene of interest. Note that the donor construct elements are not to scale. A CMV promoter is depicted as an example of an exogenous promoter. HA-L, HA-R; left and right homology arms, respectively. FP; fluorescent protein. CMV; CMV promoter. Created with BioRender.com.

Several other groups have designed selection techniques where a selectable gene is incorporated directly into the donor DNA for modifying the GOI. The Porteus lab has developed several strategies enabling the selection of precisely edited cells, working on the clinically relevant human haematopoietic stem cells (HSCs) [51,52]. For example, Bak et al. described a method where foreign selection cassettes are combined in a donor DNA for modifying a GOI. In this case, the donor DNA is provided by rAAVs (recombinant adeno-associated viruses), and an exogenous promoter drives the expression of the selectable gene [51]. The advantage here is that strong selection can be applied, and expression of the selectable gene is not dependent on the promoter of the GOI. However, as with the method above, there is a limitation as to where the mutation can be made if the general genomic structure is maintained. To circumvent this problem, they implement a construct where a modified cDNA of the GOI is inserted along with the selection cassette. This enables mutagenesis of the GOI at any location, rather than being limited to modification at the N- or C-termini. This method provides for the expression of the modified GOI from its native promoter, but the genomic structure is no longer native [51]. Furthermore, as with eFlut, the modified cells express foreign genes. In another application of marker gene selection, Scheller et al. used two donor DNAs with different fluorescent protein-expressing selection cassettes to interrupt the *CCR5* gene, a co-receptor for HIV-1, making the cells resistant to HIV-1 infection [53]. Selecting for double-positive cells ensures that the gene editing is biallelic. Another method, GEIS (Gene Editing through an Intronic Selection marker), provides a means for selecting the desired mutation that is not necessarily localized to the gene termini [54]. Here, the donor DNA is designed to insert a foreign selection gene and promoter into an intron close to the desired editing site of the GOI. The foreign gene cassette may need to be removed later in a second step if it interferes with expression of the GOI. These strategies are conceptually summarized in Figure 2.

### 3.3. Selection Involving Co-Editing

As described above, the HDR pathway is not active in most cells; therefore, cells competent for this type of editing are in the minority. However, within a single HDR-competent cell, multiple editing events can occur simultaneously [4]. If a gene of interest is edited simultaneously with a selectable marker, both requiring HDR for their editing, then cells that survive the selection should be enriched for editing in the GOI. Properly editing the selectable gene demonstrates that the cell was competent for HDR-dependent editing. The level of enrichment would still be contingent on the editing efficiency of each of the loci. Yan et al., in a study described below, compared different guides for the selectable gene, finding that, in general, the best co-editing results were achieved with the strongest guide for the selectable gene [55]. However, as there were some differences in editing success among the target GOIs, this does suggest that adjusting the guide efficiencies between the selectable gene and GOI may improve the outcome. Furthermore, it may be beneficial to increase the amount of the gRNA targeting the GOI relative to the selectable guide, although this must be confirmed. Shy et al. analyzed co-incidental insertion (COIN) efficiency, comparing donor DNA for the selectable gene that was less optimal (short homology arms) vs. more optimal (long homology arms) [56]. When using the less optimal donor for the selectable gene, the enrichment of editing in the GOI was much higher. This is consistent with the model that co-editing, COIN, works because it selects those cells that were most proficient at HDR from the total population. 

**Figure 3:**
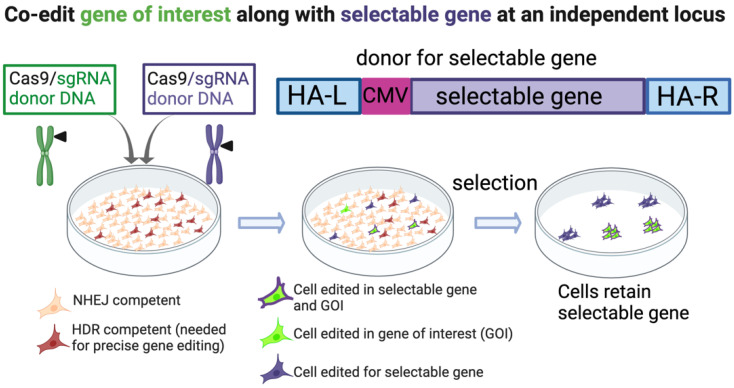
Selection marker co-edited with gene of interest. The selectable gene is edited into a locus independent of the gene of interest. Note that the donor construct elements are not to scale and depict a general approach. In this example, a CMV promoter is used to drive expression of the selectable gene. HA-L, HA-R; left and right homology arms, respectively. Created with BioRender.com.

A simple application of the co-editing strategy is to insert a selectable gene, such as a fluorescent protein or antibiotic resistance, into a chosen genomic locus while simultaneously editing the GOI [56,57,58] (Figure 3). The advantages of this strategy are that the selection can be strong since a suitable endogenous promoter can be chosen for targeting that does not necessarily have to be associated with the GOI. Alternatively, a strong exogenous promoter can be used to drive the cassette. When using this system, it is important to know whether modification of the locus used for selection affects the phenotype of the targeted cells, both in the case of the insertion of the selective gene, and if one of the alleles has been mutated (indels) due to repair of the DSB by NHEJ. Furthermore, in any strategy where multiple chromosomes are cut, it is important to verify that unwanted translocations have not occurred [59]. 

## 4. Scarless and Semi-Scarless Selection

In the selection methods described above, the resulting cell lines express a foreign gene. However, there are situations where such a modification is undesirable. Ideally, we would like to make a mutation in the GOI without perturbing anything else in the genome—that the editing should be “scarless”. The optimal situation is when the modified GOI itself provides a selection strategy. This was demonstrated by Roth et al., where primary human T cells were edited to repair a genetic defect that prevented the expression of IL-2α receptor, or alternatively, to introduce a unique T cell receptor recognizing a tumor antigen [35]. In these cases, the modified cells express unique cell surface proteins and can be isolated by FACS. 

### 4.1. Two Step Editing—Insert, then Remove, Selectable Marker

If a foreign gene is used as a selectable marker, and yet we require the final cell product to be “scarless”, a solution is to perform two rounds of editing, the first where the desired mutation is inserted in the GOI along with co-editing of a selectable gene, followed by a second round of CRISPR editing where the selectable gene is removed, also employing a positive-negative selection strategy [60]. Ikeda et al. used this method to create scarless mutations in human pluripotent stem cells. For selective markers, they used mCherry-T2A-tCD19 (truncated CD19). This enabled selection of cells using magnetic beads assisted cell sorting with an antibody to CD19. Moreover, the level of mCherry expression enabled isolation of biallelically edited cells via FACS. The selectable markers were then removed in a second CRISPR step. Provided that both editing steps are efficient and do not create unwanted mutations, this method can produce cells that are edited exclusively in the GOI. However, the need for two steps reduces the yield of the properly edited cells and increases the time required to make them. In addition, it is conceivable that cells could lose expression of the selectable marker in the second step through knockout due to indels rather than precise editing, although this did not occur in the examples presented [60]. Isolated clones must be screened to ensure that the editing was in fact scarless. 

**Figure 4:**
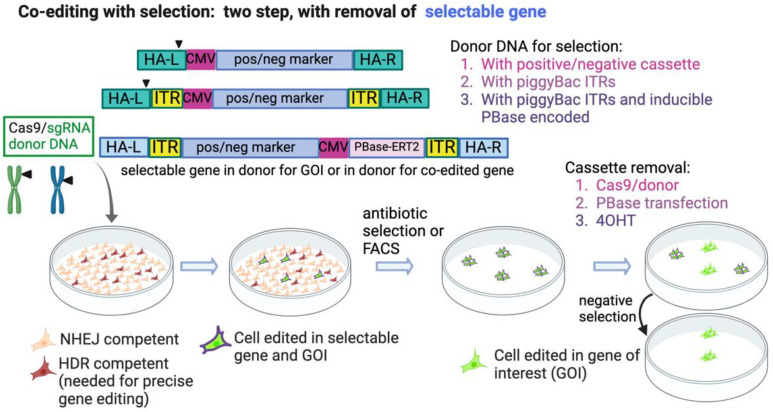
Two-step scarless editing with removal of the selectable gene. This figure is based on strategies presented in references [56,60,62,63,64,65,67], although the precise constructs are not fully reproduced, and the depictions are not to scale. The cassettes encode positive and negative selectable genes, depicted here in a general scheme driven by a CMV promoter. The selectable cassettes are contained within the donor DNA for editing the GOI or are included in a donor DNA for co-editing a locus independent of the GOI. HA-L, HA-R; left and right homology arms, respectively. ITR; inverted terminal repeats for later removal of cassette via piggyBac transposase. PBase-ERT2; PBase fused to modified ERT2 for later induction by tamoxifen (4OHT). Created with BioRender.com.

Alternatively, several groups have taken advantage of the piggyBac system [61] to seamlessly remove selectable markers. The Kan group incorporated this technology into CRISPR-Cas9 editing [62,63], using it to create specific mutations in human iPSCs. Ye, et al. generated iPSCs homozygous for the naturally occurring CCR5D32 that renders cells resistant to HIV infection [62]. The donor DNA construct contained a Puro-TK-Neo cassette flanked by the TTAA recognition sites for the piggyBac transposase. The TTAA site is present naturally close to the site targeted in *CCR5*, and thus removal of the cassette following selection by transfection of piggyBac transposase (PBase) restores the original TTAA sequence and results in scarless editing. Following cassette removal, the cells are screened for random integrations of the donor plasmid using negative selection with FIAU and verified by Southern blotting. Because the piggyBac transposase used here is integration-competent, reintegration of the transposon catalyzed by piggyBac transposase occurs in 90% of cells, thus reducing the yield of precisely edited cells. Nonetheless, two biallelic clones with no piggyBac remnants were produced, and these were differentiated into macrophages/monocytes that were resistant to HIV infection [62]. Using a similar strategy, Xie et al. corrected the defect in the *HBB* gene in iPSCs derived from a patient with β-thalassemia [63], and Kondrashov et al. similarly edited cardiac-associated genes in human pluripotent stem cells [64]. Shy et al. named their co-editing technique COIN (co-incidental insertion) and used it to select HDR-dependent edited mouse ES cells. They expanded this technique to incorporate a donor selection cassette named pRIND, for PBase-Removable Insertion DNA [56]. An advantage to this cassette is that it also encodes the PBase transposase that is required to seamlessly remove the cassette after selection, avoiding the extra step of a separate transfection of PBase. To prevent the transposase from acting immediately, it is fused to a modified estrogen receptor, ERT2, which prevents it from entering the nucleus. Following selection, 4-Hydroxy Tamoxifen (4OHT) is added to stimulate nuclear translocation of PBase, and the excision of the cassette. These works demonstrated the ease and versatility of the Cas9/piggyBac system to efficiently edit cells and seamlessly remove a selection cassette, but they had the problem of reintegration of the piggyBac transposon. Singh et al. used a similar selection cassette with a negative selection module to engineer a point mutation in the *MLL2* gene [65]. However, rather than use the transposase capable of mediating insertion, they employed an excision-only piggyBac transposase [66], thus preventing non-specific insertions of the transposon. Arias-Fuenzalida et al. developed a further advance of this technique, which they termed FACE, for FACS- assisted CRISPR-Cas9 editing [67]. In this application, two donors, each harboring a different fluorescent protein, are used in targeting the cells. FACS sorting for double-positive cells ensures that biallelic editing was achieved. The donor vectors also have a negative selection module to select against random integration of the donor vector. This innovation greatly improves the recovery of biallelically edited cells. To remove the selectable markers, cells are transfected with an mRNA encoding a codon-optimized hyperactive and excision-only variant of the piggyBac transposase [66,68]. This application is ideal for targeting GOIs that can tolerate the temporary interruption by the selection cassette, have a nearby TTAA site, or be silently introduced. These methods are summarized in Figure 4. 

### 4.2. Co-Editing of an Endogenous Gene for Selection 

**Figure 5:**
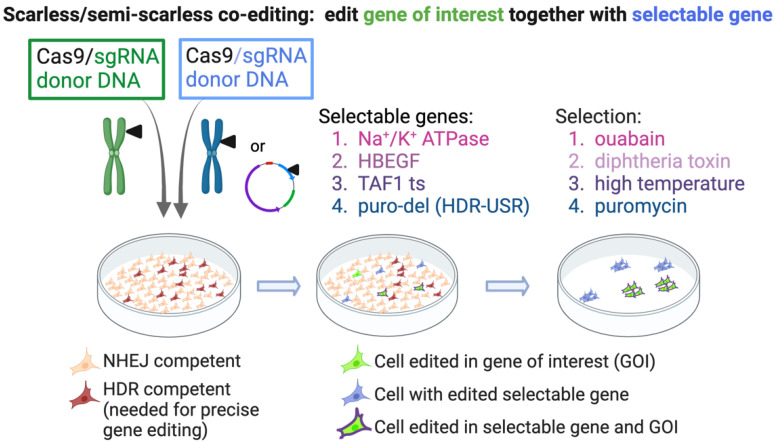
Co-editing an endogenous or plasmid-encoded selectable gene. Cells are transfected with Cas9/donor for the GOI, in addition to Cas9/donor for an endogenous gene, or with the HDR-USR reporter plasmid. The co-edited genes provide resistance to selection, and as the genomic perturbations are minimal, these methods are scarless or semi-scarless. Created with BioRender.com.

The next category of scarless selection involves co-editing an endogenous gene to allow survival in the presence of a drug or metabolic stress (Figure 5). The advantage to using an endogenous gene is that the conditions for editing may be more similar to that of the GOI, and thus the enrichment will be higher for cell editing in the GOI. Furthermore, the donor DNA may be an ssODN, obviating the need for plasmid or viral vectors. For example, Agudelo et al. mutagenized the sodium/potassium pump (Na^+^/K^+^ ATPase), rendering the cells resistant to ouabain [69]. The mutations engineered to allow ouabain escape are naturally occurring in metazoans, and the mutated ATPase functions normally. This approach was useful for improving the yield of coselected mutations in the GOI in cell lines as well as in primary human cells, with *HBB* being among the genes targeted. Using a similar approach, named “Xential”, Li et al. targeted the diphtheria toxin receptor, heparin-binding EGF-like growth factor (HBEGF), allowing the cells to survive selection with diphtheria toxin (DT) [70]. The DT-selected cells are edited biallelically at this locus since cells with a WT allele do not survive the selection, and only biallelically edited cells are recovered. The advantage of these methods is that the conditions for editing the endogenous selectable gene are likely to be similar to editing the gene of interest, meaning that the enrichment achieved with selection should be high. In addition, the editing can be performed in the absence of plasmids; the Cas9/gRNA can be delivered as a ribonucleoprotein complex, and an ssODN can serve as donor. The disadvantage is that the method is only “semi-scarless”, since the selectable gene is edited permanently. In some applications, these mutations could affect cell phenotype. 

Another approach is to use a temperature-sensitive (ts) mutation in an essential gene as the selectable marker. HDR-dependent repair of the ts mutation allows the properly edited cells to survive selection at high temperature. Since the mutated gene is restored to wild-type sequence, this selection is theoretically “scarless”. Co-editing of a ts mutation in TAF1, the major subunit of the basal transcription factor complex TFIID can produce high yields of cells edited in the GOI [71]. The *TAF1* gene is present on the X-chromosome, and for cell lines with more than one X chromosome (such as HEK293, which has three), the ts clones most easily obtained have one ts allele, with the others being knocked out due to indels. The knocked-out alleles mean that this method is only semi-scarless since these alleles will remain knocked out downstream. However, this situation has certain advantages because the selectable gene has only one allele that can be targeted in the co-editing strategy. Because only one allele is targeted, it prevents any possibility of chromosomal translocations with this locus during the co-editing of a GOI. Furthermore, since one copy of *TAF1* is sufficient for cell survival (as in male cell lines), the knock-out of the non-ts alleles is not detrimental. Because the ts cell line is generated and characterized ahead of the co-editing step, all of the downstream cell lines produced using it will be isogenic for the ts gene locus. In other applications where an endogenous gene is targeted as a selectable marker, unless the editing of the selectable locus is precise and biallelic, each co-editing event will generate different genotypes at the locus of the selectable gene, meaning that the resulting cell lines will not be isogenic.

### 4.3. Scarless Selection—By Editing a Plasmid-Based Marker

A simple and fully scarless approach was demonstrated by Yan et al. [55]. This method, termed “HDR-USR” (for universal surrogate reporter), relies on the repair of a reporter-containing plasmid (Figure 5). The HDR-USR plasmid encodes Cas9, a mutated antibiotic resistance gene, a donor fragment of the mutated gene, and a gRNA specific to the mutated gene. Cells that successfully repair the selective gene survive the antibiotic selection. Because the gRNA is unique to the mutated foreign gene, this selection strategy does not require the host genome to be cut at a site other than the GOI, precluding the danger of translocation. Following selection for a limited time, the selection plasmid is not integrated into the genome and is lost through dilution after several passages. The cells are then once again sensitive to the antibiotic, and the same HDR-USR reagent may be used for subsequent rounds of mutagenesis. 

Although base editing and prime editing do not have the same constraints as DSB-dependent editing, such as cell cycle dependence on endogenous DNA repair proteins, it is still helpful to select cells in which editing was carried out with fidelity. For this reason, screening reporter/selection plasmids have been developed for both of these methods that are conceptually similar to HDR-USR. Adenine and Cytosine BaseEditing Antibiotic Resistance Screening Reporter (ACBE-ARSR) [72] improves the efficiency of ABE and CBE by 1.9 and 4.6-fold, respectively, with editing efficiencies reaching 90%. PEAR (prime editor activity reporter) is a fluorescent tool for identifying single cells with prime editing events, and its use can increase the edited population by up to 84% [73]. 

## 5. Perspectives

Thanks to CRISPR, it is now possible to create mutant cell lines in the laboratory and to envision specific gene therapy treatments in the clinic that were difficult or impossible to achieve a decade ago. However, there are still stumbling blocks to overcome to improve the quality and efficiency of cell editing, particularly for clinical use. The ideal end-product is a cell that is edited only in the GOI, with neither transgenes nor unwanted mutations present. To prevent the possibility of unwanted integrations of foreign DNA, the Cas9/gRNA should ideally be delivered as a ribonucleoprotein complex, and the donor DNA should not randomly integrate. Thus, ssODN donor DNAs would likely be the safest choice, although these are limited to editing small genomic changes. Where appropriate, the non-DSB-causing methods of base editing and prime editing can be used, with the advantage that they will not lead to undesired off-target DSBs or the improper editing of the on-target DSB. These methods may suffice for applications requiring small genomic changes, yet there are others in which plasmid, linear dsDNA fragments, or viral vectors such as rAAV may be the only recourse for encoding the donor template. Methods combining strategies, for example, restriction of Cas9 expression, and recruitment of HDR-promoting factors, can together improve the quality and yield of edited cells. However, even with these enhancements, the edited cells often represent a low percentage of the total population and must be isolated and characterized. For this reason, selection strategies, as described above, should be considered since they offer high rates of enrichment, even up to 100%, depending on the system. Many of the methods above employ selectable transgenes, which can be very useful in the basic research setting but may not always be appropriate for the clinic. Methods offering scarless selection of knock-in cells provide ways to circumvent this issue, but they also come with their own limitations. Firstly, most of these protocols involve several steps, which lowers the overall yield of positive cells. Secondly, some of these interim steps involve an interruption of the GOI, which may not always be tolerated. Thirdly, some of the methods are only semi-scarless, leaving behind a small but potentially significant genomic change. It is clear that each method has its advantages and disadvantages, and thus the benefits must be weighed against the potential damage in each case.

With all methods, there exists the possibility of unwanted genomic changes, whether they are due to the off-target activity of Cas9, illegitimate integration of plasmid or other donor DNA, or translocations between different cut chromosomes. CRISPR-Cas9-produced DSBs can lead to large deletions, extending over many kilobases, which can often be missed by screening methods using PCR to focus on the local targeted region [74]. In addition to unwanted indel mutations, DSBs can lead to aneuploidy, with severe effects on genome stability and carcinogenesis. A recent study of genome editing outcomes in primary human T cells found that aneuploidy and chromosomal truncations are frequent outcomes of genome editing in these cells [75]. CRISPR edited cells for therapeutic use should therefore be thoroughly screened for their genomic integrity. The use of selection strategies that enrich productively edited cells can be instrumental for isolating the rare cells precisely edited at the GOI, reducing the number of colonies that must be screened and characterized. A summary of the selection strategies described is presented in Table 1.

## Author Contributions

Writing—original draft preparation, N.R.; writing—review and editing, Y.S. and N.R. All authors have read and agreed to the published version of the manuscript.

## Figures and Tables

**Table 1:** Selection strategies for CRISPR-edited cells.

Selection Strategy	Description	Scarless?	Reference
Marker linked to GOI	eFlut plasmid sets for generating dsDNA donors with selectable genes by PCR- for tagging N- or C-termini of endogenous genes	No	[50]
	Foreign selection cassettes are combined in a donor DNA for modifying a GOI	No	[51]
	Two donor DNAs with different fluorescent protein-expressing selection cassettes for selection of biallelically edited cells	No	[53]
	GEIS (Gene Editing through an Intronic Selection marker)	Yes, if cassette does not interfere with splicing or GOI expression	[54]
Co-editing with independent locus	Co-editing of GOI alongside selectable gene requiring HDR	No	[57,58]
	COIN (co-incidental insertion)	No	[56]
Scarless selection	Gene edit is itself selectable: FACS sorting for TCR in primary human T cells	Yes	[35]
Two-step scarless selection: insert, then remove selectable marker	Two CRISPR editing steps—first step CRISPR to introduce selectable marker and edit GOI, second CRISPR to remove selectable marker	Yes	[60]
Selectable cassette flanking mutation in GOI; removal using piggyBac transposase	Co-editing of selectable cassette followed by removal by transfected piggyBac transposase	Yes, if cassette not re-inserted by transposase	[62,63,64]
	Co-editing with removable selection cassette expressing PBase-ERT2 for induction of cassette removal with 4OHT	Yes, if cassette not re-inserted by transposase	[56]
	Editing of GOI with donor containing removable selection cassette. Removal via transfection with excision-only PBase	Yes	[65]
	Two excisable fluorescent donor cassettes to permit sorting of biallelic clones, removal of cassettes with excision-only PBase	Yes	[67]
Co-editing with selectable endogenous gene	Co-editing of sodium/potassium pump for resistance to ouabain	Semi	[69]
	Co-editing of HBEGF for resistance to diphtheria toxin, “Xential”	Semi	[70]
	Co-editing of TAF1ts, selective gene restored to wt sequence	Semi	[71]
Co-editing with plasmid-encoded selectable gene	“HDR-USR” (universal surrogate reporter) Truncated puro-resistance gene is corrected on plasmid	Yes	[55]
	“ACBE-ARSR” Adenine and cytosine base editing antibiotic resistance screening reporter	Yes	[72]
	“PEAR” Prime editor activity reporter	Yes	[73]

## Data Availability

Not applicable.
